# Trustworthy guidelines – excellent; customized care tools – even better

**DOI:** 10.1186/s12916-015-0436-y

**Published:** 2015-09-01

**Authors:** Glyn Elwyn, Casey Quinlan, Albert Mulley, Thomas Agoritsas, Per Olav Vandvik, Gordon Guyatt

**Affiliations:** The Dartmouth Institute for Health Policy and Clinical Practice, 37 Dewey Field Road, Hanover, NH 03755 USA; The Mighty Mouth, Mighty Casey Media, 9101 Patterson Avenue Suite 57, Richmond, VA 23229 USA; Department of Clinical Epidemiology and Biostatistics, McMaster University, Faculty of Health Sciences, Hamilton, Ontario Canada; Department of Health Management and Health Economics, Institute of Health and Society, University of Oslo, Norway, Oslo Norway

**Keywords:** Clinical practice guidelines, Evidence based medicine, Patient engagement, Shared decision making

## Abstract

**Background:**

The ability to do online searches for health information has led to concerns that patients find the results confusing and that they often lead to expectations for treatments that have little supportive evidence. At the same time, the science of summarizing research evidence has advanced to the point where it is increasingly possible to quantify treatment tradeoffs and to describe the balance between harms and benefits for individual patients.

**Discussion:**

Trustworthy clinical practice guidelines provide evidence-based recommendations to health care practitioners based on assessments of study-level averages. In an effort to customize the use of evidence and ensure that choices are consistent with their personal preferences, tools for patients have been developed. Gradually, there is recognition that the audience for high quality evidence is much wider than merely health care professionals – and that there is a case to be made for creating tools that translate existing evidence into tools to help patients and clinicians work together to decide next steps.

**Summary:**

We observe two processes occurring: first, is the recognition that decision making in healthcare requires collaboration and deliberation, and second, to achieve this, we need tools designed to customize care at the level of individuals.

## Background

### Forbidding Dr. Google

In November 2014, the Flemish government paid the Belgian Center of Evidence-Based Medicine to create a video telling patients to avoid Google before going to see their own doctor [1]. The reaction in social media was predictable. It is too late to turn the tide. Patients visit Google in increasing numbers: a phenomenon that is not going to be influenced by one media campaign. Yet, the underlying concern is valid enough. It is very difficult, if not impossible, to interpret the results of such searches – where are the data we can trust, who generated the information, and where do the conflicts of interest lie? Which of the thousands of URLs listed have any degree of transparency about who generated the information, how did they do so, and what do they stand to gain in some way by its use? To what extent can patients rely on the experience of other patients or be directed to good summaries of scientific evidence? Should Google algorithms prioritize recognized sources of high quality evidence summaries?

It was 40 years ago when Archie Cochrane wrote: “It is surely a great criticism of our profession that we have not organized a critical summary, by specialty or subspecialty, adapted periodically, of all relevant randomized control trials [2].” This observation led Iain Chalmers to propose the Cochrane Collaboration, which has since pioneered methods to produce high quality systematic reviews. These systematic reviews have become the cornerstones of evidence-based medicine and have been increasingly used to develop clinical practice guidelines [3]. Until recently, however, the majority of guidelines were not rigorously developed. They were – among many other limitations – compromised by the conflicted interests of their panelists [4, 5]. Fortunately, developments that include clear standards for guidelines and innovative strategies to deal with conflict of interest and appropriately engage patients in the process are resulting in evidence that is increasingly trustworthy [6, 7] – there is now more confidence that the synthesis represents the most rigorous approach to interpreting existing science.

However, guidelines have limitations, especially when we consider the necessity to customize treatments and decisions. It is not only difficult to find guidelines, it is also difficult to know which ones are of high quality. Guidelines can only make recommendations for typical patients, and cannot ensure that individual values and preferences are accounted for in the ultimate management decisions.

### Going beyond clinical practice guidelines

Despite the enormous investment in guideline production, only a limited number have been successfully tailored so that they are optimally useful to clinicians and very few, if any, have been tailored for optimal usefulness to patients. Clinicians have the task of tailoring evidence to individuals – to people at different ages and with different sets of problems, concerns, preferences, and priorities. Many existing clinical practice guidelines do not support this task despite recent efforts to include patients as stakeholders in guideline development [8, 9], and no conventional guideline can fully support this task.

There have been efforts to make clinical guidelines more accessible to a wider audience, typically by making them shorter and easier to read. Others want to create more recognizable knowledge sources for patients (Table 1). For example, the Wiki Project Med, working with the Cochrane Collaboration and Wikipedia, has been recently launched with the aim of giving “*free access to the sum of all medical knowledge*” [10]. However, the production of evidence summaries for patients is at a very early stage and the public is barely aware such resources exist [11]. Nevertheless, creating wider access will not address the core problem.

**Table 1:** Evidence designed for the public: examples of existing efforts

Organizations	Activity
Guidelines International Network (GIN)	GIN provides advice on how to develop public versions of guidelines, giving details and guidance about the process [27]
Choosing Wisely collaboration with Consumer Reports, USA	Produces evidence-based material that is in part public-facing [28]
Consumers United for Evidence-Based Healthcare (CUE)	Some specialty groups in the USA collaborate with CUE to produce public-facing versions of guidelines, and involve patients in the development process
The Developing and Evaluating Communication Strategies to Support Informed Decisions and Practice Based on Evidence Collaboration (DECIDE) Funded by the European Commission 7th Framework	DECIDE has a work stream on dissemination strategies for clinical guidelines that are public-facing [29]
The National Institute for Health and Care Excellence (NICE) UK	NICE, as well as involving patients as stakeholders, produces plain English versions of patient guidelines [30]
Scottish Intercollegiate Guidelines Network (SIGN)	SIGN produces public-facing versions of clinical guidelines on their website [31]

Guidelines that summarize scientific evidence and provide recommendations that apply at a general level are necessary but far from sufficient. Nevertheless, however much we work to make guidelines readable and accessible to a wider audience, they will be limited in their application to the situations – likely a majority – that are sensitive to patients’ differing circumstances, preferences, and priorities. Additional tools are necessary to ensure that guidelines are optimally applicable – tools that are just as rigorous when it comes to evidence but that also help individualize decisions to the patient at hand. Such tools would compare treatments and make explicit the tradeoffs between the benefits and harms. Such tools exist, and have been termed patient decision support.

### Different types of tools

Less well known than guidelines, patient decision support tools or aids have been developed over the last few decades. Their goal is to support patients to compare alternative treatment options. Some tools are designed for use by patients before clinical encounters and other much shorter versions are designed for use by patients and clinicians together [12, 13]. A systematic review has shown that these tools have a positive impact on patient knowledge and risk perception, and often result in significant shifts in choice of treatment [14]. Despite their benefits, these interventions have not been widely implemented into clinical practice [15].

Many of these tools also include information about the experiences of patients, and describe what it is like to have the illness and to experience the treatment, including side effects. Patients and their families find this kind of information highly valuable, and as important as understanding the probabilities of likely outcomes. An excellent example of this kind of information is the Healthtalk Online website [16–18]. Patient decision tools have been widely advocated for facilitating shared decision making, in which clinicians strive to ensure their patients are aware of the best evidence available, before eliciting their preferences. These tools help take evidence-based medicine to its logical endpoint, to individual end-users, where decisions are customized not only to the characteristics of the individual being treated but also aligned with their priorities.

## Discussion

### Towards customized care tools

Whilst we can confidently make the argument for the adoption of evidence-based tools to help produce customized care for individuals, there are many steps required before this vision becomes a reality. Evidence synthesis will become more efficient, automated, and will increase the speed at which tools can be populated with risk data that can be used at the level of individuals. There is considerable research published on how to produce these types of tools and about how to communicate risk that it is understood well by both clinicians and patients. There are also sources that provide patients and their families with information about illness and treatment experience. Bringing these elements together will be a challenge, and there will be a need to have versions that can be viewed or used independently as well as versions that are brief enough for use in clinical encounters.

How to make these widely accessible is going to be another significant hurdle. Clinical practice guidelines have been made available to clinicians by a range of different arrangements. Patient-facing decision support tools are not available in the same way, they are available commercially by a small number of producers, or are part of research evaluations. Patients and their families cannot easily find them using Internet search engines.

Here, then, are some steps that would help us achieve a future where evidence-based customized care tools might become more available.

#### Efficient evidence processing

The work required to locate the highest quality evidence before producing the best estimates of treatment effects, burden [19], benefits, and harms takes time and expertise. The methods have, after 40 years of work, been well developed. The next steps being considered are how to best automate the process in order to generate faster data to populate different types of tools.

#### Sharing experiences

Patients are, of course, interested in more than evidence-based estimates of risk. They want to know exactly what they will face as they develop an illness, and what do other patients say about their experiences of illness. What are their main concerns, questions, and priorities [20, 21]? Researchers working with Healthtalk Online in the UK have summarized the illness experience of many patients with many conditions [16–18]. More work is required on how best to integrate this information into tools that can help customize care.

#### Tool production

**Fig. 1:**
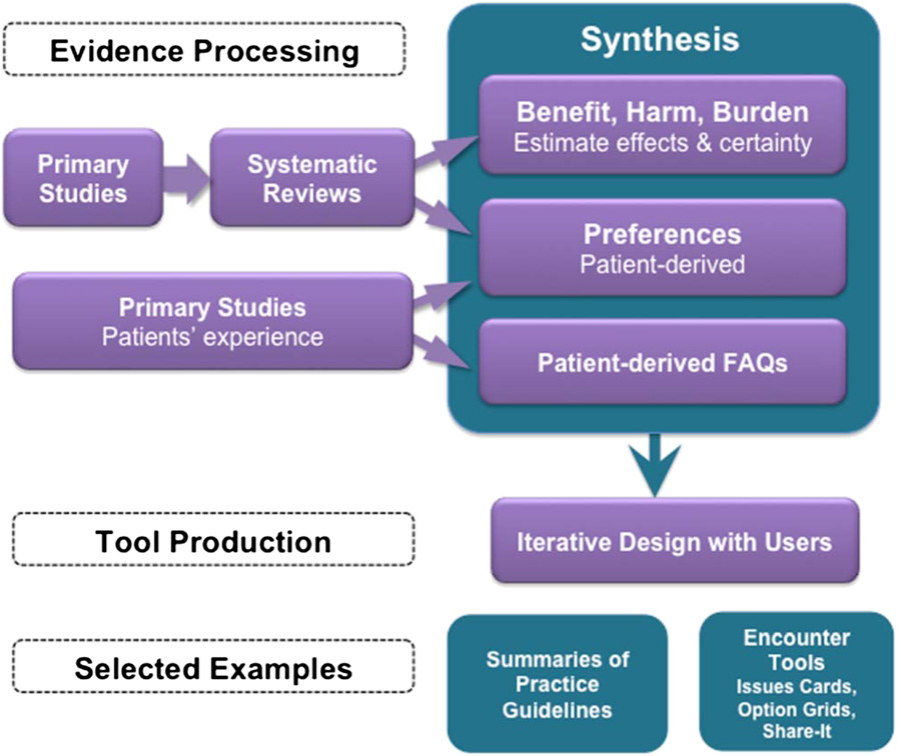
From evidence synthesis to customized care tool production

As mentioned, different types of tools will be necessary to facilitate shared decision making. What ‘matters most to patients’ should guide the presentation of evidence, specifically in terms of organizing the content and conveying the probabilities of treatment effects, harms, and burden in ways that communicate risk most effectively. Short tools such as Issue Cards [12], Option Grids [13], and SHARE-IT tools [22] have been designed to facilitate collaboration and deliberation in clinical encounters [23]. They do this by being brief and available for both patient and clinician to use together, either on paper or tablets. Research efforts are now addressing usability of tools for the clinical encounter [7, 22, 24]. We argue for a future state where patients and clinicians rely on the same evidence base – and on tools that are used collaboratively – to get to consensus on treatment, where that is possible. We are working, in collaboration with many others, to systematize evidence synthesis to facilitate the production of trustworthy tools, for multiple purposes, that can be widely shared (Fig. 1).

## Summary

### Next steps

The three areas described above – evidence synthesis, patient experience, and patient-facing tool production – have stemmed from independent areas of work. There are, however, signs of increasing overlap between the groups. A joint conference focusing on shared decision making and evidence-based healthcare will be held in Australia in 2015 [25]. Many from the Society for Participatory Medicine, one of many patient-led organizations, have attended relevant conferences and are advocates for these evidence-based patient-facing tools, and Casey Quinlan, a prominent member, is a co-author on this article.

Progress will depend on advances in all these areas if we are to ensure that trustworthy evidence can be used collaboratively in clinical encounters [23], with clinicians willing and able to achieve shared decision making with patients (Box 1). Such tools would include patients in the development process, and would move away from the view that medicine has to be determined solely by ‘what is medically best’ and allow patients’ priorities, concerns, and preferences to be considered as well [26]. It is time to move beyond the limitations of current clinical practice guidelines and focus our energy on tools that will help facilitate customized care at the level of individuals and their families.

## Box 1 Steps towards customized care tools

- Create trustworthy evidence – in addition to applying new standards and systems for evidence synthesis, further work should focus on optimal presentation and dissemination strategies, and a commitment to create public-facing versions with optimal patient engagement strategies
- Create generic customized care tools – if patients can understand them, so will clinicians, and there is more chance that better conversations will be generated. Busy clinicians value short tools, but will be willing to read longer versions if they need to know more about the underlying evidence
- Make the tools easy to find – use search engine optimization methods to ensure the tools are highly ranked in results
- Create trusted sources – people are becoming more skeptical, we need to create trusted sources of evidence and be transparent about limitations in the quality of the evidence
- Bring tools to the clinical encounter – innovative presentation formats, based on trustworthy guidelines, designed for use in clinical encounters could support collaboration and deliberation [23]
